# Comparative genomics reveals birth and death of fragile regions in mammalian evolution

**DOI:** 10.1186/gb-2010-11-11-r117

**Published:** 2010-11-30

**Authors:** Max A Alekseyev, Pavel A Pevzner

**Affiliations:** 1 Department of Computer Science & Engineering, University of South Carolina, 301 Main St., Columbia, SC 29208, USA; 2 Department of Computer Science & Engineering, University of California, San Diego, 9500 Gilman Dr., La Jolla, CA 92093, USA

## Abstract

**Background:**

An important question in genome evolution is whether there exist fragile regions (rearrangement hotspots) where chromosomal rearrangements are happening over and over again. Although nearly all recent studies supported the existence of fragile regions in mammalian genomes, the most comprehensive phylogenomic study of mammals raised some doubts about their existence.

**Results:**

Here we demonstrate that fragile regions are subject to a birth and death process, implying that fragility has a limited evolutionary lifespan.

**Conclusions:**

This finding implies that fragile regions migrate to different locations in different mammals, explaining why there exist only a few chromosomal breakpoints shared between different lineages. The birth and death of fragile regions as a phenomenon reinforces the hypothesis that rearrangements are promoted by matching segmental duplications and suggests putative locations of the currently active fragile regions in the human genome.

## Background

In 1970 Susumu Ohno [1] came up with the Random Breakage Model (RBM) of chromosome evolution, implying that there are no rearrangement hotspots in mammalian genomes. In 1984 Nadeau and Taylor [2] laid the statistical foundations of RBM and demonstrated that it was consistent with the human and mouse chromosomal architectures. In the next two decades, numerous studies with progressively increasing resolution made RBM the *de facto *theory of chromosome evolution.

RBM was refuted by Pevzner and Tesler [3] who suggested the Fragile Breakage Model (FBM) postulating that mammalian genomes are mosaics of fragile and solid regions. In contrast to RBM, FBM postulates that rearrangements are mainly happening in fragile regions forming only a small portion of the mammalian genomes. While the rebuttal of RBM caused a controversy [4-6], Peng *et al. *[7] and Alekseyev and Pevzner [8] revealed some flaws in the arguments against FBM. Furthermore, the rebuttal of RBM was followed by many studies supporting FBM [9-31].

Comparative analysis of the human chromosomes reveals many short adjacent regions corresponding to parts of several mouse chromosomes [32]. While such a surprising arrangement of synteny blocks points to potential rearrangement hotspots, it remains unclear whether these regions reflect genome rearrangements or duplications/assembly errors/alignment artifacts. Early studies of genomic architectures were unable to distinguish short synteny blocks from artifacts and thus were limited to constructing large synteny blocks. Ma *et al. *[33] addressed the challenge of constructing high-resolution synteny blocks via the analysis of multiple genomes. Remarkably, their analysis suggests that there is limited breakpoint reuse, an argument against FBM, that led to a split among researchers studying chromosome evolution and raised a challenge of reconciling these contradictory results. Ma *et al. *[33] wrote: 'a careful analysis [of the RBM vs FBM controversy] is beyond the scope of this study' leaving the question of interpreting their findings open. Various models of chromosome evolution imply various statistics and thus can be verified by various tests. For example, RBM implies exponential distribution of the synteny block sizes, consistent with the human-mouse synteny blocks observed in [2]. Pevzner and Tesler [3] introduced the 'pairwise breakpoint reuse' test and demonstrated that while RBM implies low breakpoint reuse, the human-mouse synteny blocks expose rampant breakpoint reuse. Thus RBM is consistent with the 'exponential length distribution' test [2] but inconsistent with the 'pairwise breakpoint reuse' test [34]. Both these tests are applied to *pairs *of genomes, not taking an advantage of multiple genomes that were recently sequenced. Below we introduce the 'multispecies breakpoint reuse' test and demonstrate that both RBM and FBM do not pass this test. We further propose the *Turnover Fragile Breakage Model *(TFBM) that extends FBM and complies with the multispecies breakpoint reuse test.

Technically, findings in [33] (limited breakpoint reuse between different lineages) are not in conflict with findings in [3] (rampant breakpoint reuse in chromosome evolution). Indeed, Ma *et al. *[33] only considered reuse between different branches of the phylogenetic tree (*inter-reuse*) and did not analyze reuse within individual branches (*intra-reuse*) of the tree. TFBM reconciles the recent studies supporting FBM with the Ma *et al. *[33] analysis. We demonstrate that data in [33] reveal rampant but elusive breakpoint reuse that cannot be detected via counting repeated breakages between various pairs of branches of the evolutionary tree. TFBM is an extension of FBM that reconciles seemingly contradictory results in [9-31] and [33] and explains that they do not contradict to each other. TFBM postulates that fragile regions have a limited lifespan and implies that they can migrate between different genomic locations. The intriguing implication of TFBM is that few regions in a genome are fragile at any given time raising a question of finding the currently active fragile regions in the human genome.

While many authors have discussed the causes of fragility, the question what makes certain regions fragile remains open. Previous studies attributed fragile regions to segmental duplications [35-38], high repeat density [39], high recombination rate [40], pairs of tRNA genes [41,42], inhomogeneity of gene distribution [7], and long regulatory regions [7,17,26]. Since we observed the birth and death of fragile regions, we are particularly interested in features that are also subject to birth and death process. Recently, Zhao and Bourque [38] provided a new insight into association of rearrangements with segmental duplications by demonstrating that many rearrangements are flanked by *Matching Segmental Duplications *(MSDs), that is, a pair of long similar regions located within a pair of breakpoint regions corresponding to a rearrangement event. MSDs arguably represent an ideal match for TFBM among the features that were previously implicated in breakpoint reuses. TFBM is consistent with the hypothesis that MSDs promote fragility since the similarity between MSDs deteriorates with time, implying that MSDs are also subjects to a 'birth and death' process.

## Results and Discussion

### Rearrangements and breakpoint graphs

**Figure 1:**
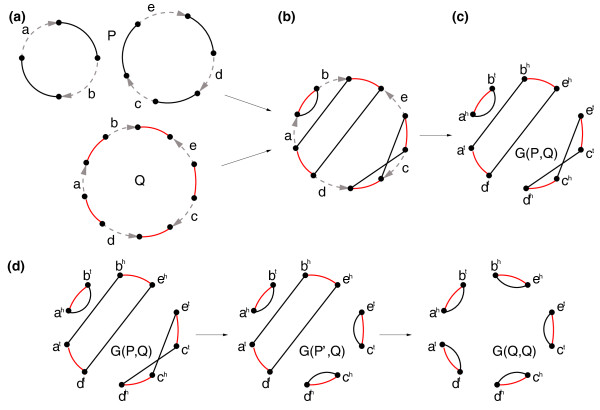
**An example of the breakpoint graph and its transformation into an identity breakpoint graph**. **(a) **Graph representation of a two-chromosomal genome *P *= (+*a *+ *b*)(+*c *+ *e *+ -*d*) as two black-obverse cycles and a unichromosomal genome *Q *= (+*a *+ *b - e *+ *c - d*) as a red-obverse cycle. **(b) **The superposition of the genome graphs *P *and *Q*. **(c) **The breakpoint graph *G*(*P*, *Q*) of the genomes *P *and *Q *(with removed obverse edges). The black and red edges in *G*(*P*, *Q*) form *c*(*P*, *Q*) = 2 non-trivial black-red cycles and one trivial black-red cycle. The trivial cycle (*a^h^*, *b^t^*) corresponds to a common adjacency between the genes *a *and *b *in the genomes *P *and *Q*. The vertices in the non-trivial cycles represent breakpoints corresponding to the endpoints of *b*(*P*, *Q*) = 4 synteny blocks: *ab*, *c*, *d*, and *e*. By Theorem 1, the distance between the genomes *P *and *Q *is *d*(*P*, *Q*) = 4 - 2 = 2. **(d) **A transformation of the breakpoint graph *G*(*P*, *Q*) into the identity breakpoint graph *G*(*Q*, *Q*), corresponding to a transformation of the genome *P *into the genome *Q *with two 2-breaks. The first 2-break transforms *P *into a genome *P' *= (+*a *+ *b*)(+*c d - e*), while the second 2-break transforms *P' *into *Q*. Each 2-break increases the number of black-red cycles in the breakpoint graph by one, implying this transformation is shortest (see Theorem 1).

For the sake of simplicity, we start our analysis with *circular genomes *consisting of circular chromosomes. While we use circular chromosomes to simplify the computational concepts discussed in the paper, all analysis is done with real (linear) mammalian chromosomes (see Alekseyev [43] for subtle differences between circular and linear chromosome analysis). We represent a circular chromosome with synteny blocks *x*_1_,..., *x_n _*as a cycle (Figure 1a) composed of *n *directed labeled edges (corresponding to the blocks) and *n *undirected unlabeled edges (connecting adjacent blocks). The directions of the edges correspond to *signs *(strands) of the blocks. We label the *tail *and *head *of a directed edge *x_i _*as $x_{i}^{t}$ and $x_{i}^{h}$ respectively. We represent a genome as a *genome graph *consisting of disjoint cycles (one for each chromosomes). The edges in each cycle alternate between two colors: one color reserved for undirected edges and the other color (traditionally called 'obverse') reserved for directed edges.

**Figure 2:**
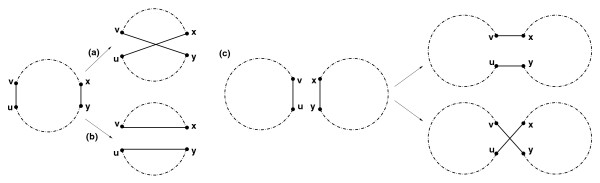
**A 2-break on edges (*u*, *v*) and (*x*, *y*) corresponding to (a) reversal, (b) fission, (c) translocation/fusion**.

Let *P *be a genome represented as a collection of *alternating *black-obverse cycles (a cycle is alternating if the colors of its edges alternate). For any two black edges (*u*; *υ*) and (*x*; *y*) in the genome (graph) *P *, we define a *2-break *rearrangement (see [44]) as replacement of these edges with either a pair of edges (*u*, *x*), (*υ*, *y *), or a pair of edges (*u*, *y*), (*υ*, *x*) (Figure 2). 2-breaks extend the standard operations of reversals (Figure 2a), fissions (Figure 2b), or fusions/translocations (Figure 2c) to the case of circular chromosomes. We say that a 2-break on edges (*u*, *x*), (*υ*, *y*) *uses *vertices *u*, *x*, *υ *and *y*.

Let *P *and *Q *be 'black' and 'red' genomes on the same set of synteny blocks X. The *breakpoint graph G*(*P*, *Q *) is defined on the set of vertices *V *= {*x^t^*, *x^h ^*| *x *∈ *χ*} with black and red edges inherited from genomes *P *and *Q *(Figure 1b). The black and red edges form a collection of alternating *black-red cycles *in *G*(*P*, *Q *) and play an important role in analyzing rearrangements (see [45] for background information on genome rearrangements). The *trivial cycles *in *G*(*P*, *Q*), formed by pairs of parallel black and red edges, represent common adjacencies between synteny blocks in genomes *P *and *Q*. Vertices of the non-trivial cycles in *G*(*P*, *Q*) represent *breakpoints *that partition genomes *P *and *Q *into (*P*, *Q*)-synteny blocks (Figure 1c). The 2-*break distance d*(*P*, *Q*) between circular genomes *P *and *Q *is defined as the minimum number of 2-breaks required to transform one genome into the other (Figure 1d). In contrast to the genomic distance [46] (for linear genomes), the 2-break distance for circular genomes is easy to compute [47]:

**Theorem 1 ***The *2-*break distance between circular genomes P and Q is d*(*P*, *Q*) = *b*(*P*, *Q*) - *c*(*P*, *Q*)*, where b*(*P*, *Q *) *and c*(*P*, *Q*) *are respectively the number of *(*P*, *Q*)-*synteny blocks and non-trivial black-red cycles in G*(*P*, *Q*).

### Inter- and intra-breakpoint reuse

**Figure 3:**
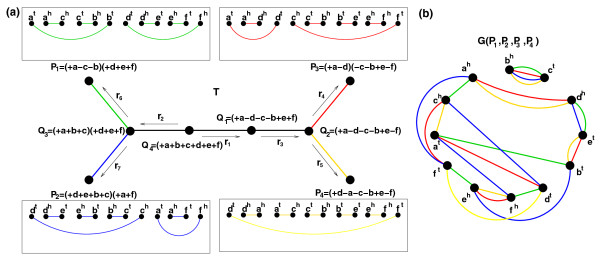
**An example of four genomes with a phylogenetic tree and their multiple breakpoint graph**. **(a) **A phylogenetic tree with four circular genomes *P*_1_, *P*_2_, *P*_3_, *P*_4 _(represented as green, blue, red, and yellow graphs respectively) at the leaves and specified intermediate genomes. The obverse edges are not shown. **(b) **The multiple breakpoint graph *G*(*P*_1_, *P*_2_, *P*_3_, *P*_4_) is a superposition of graphs representing genomes *P*_1_, *P*_2_, *P*_3_, *P*_4_.

Figure 3 shows a phylogenetic tree with specified rearrangements on its branches (we write *ρ *∈ *e *to refer to a 2-break *ρ *on an edge *e *). We represent each genome as a genome graph (that is, a collection of cycles) on the same set *V *of 2*n *vertices (corresponding to the endpoints of the synteny blocks). Given a set of genomes and a phylogenetic tree describing rearrangements between these genomes, we define the notions of inter- and intra-breakpoint reuses. A vertex *υ *∈ *V *is *inter-reused *on two distinct branches *e*_1 _and *e*_2 _of a phylogenetic tree if there exist 2-breaks *ρ*_1 _∈ *e*_1 _and *ρ*_2 _∈ *e*_2 _that both use *υ*. Similarly, a vertex *υ *∈ *V *is *intra-reused *on a branch *e *if there exist two distinct 2-breaks *ρ*_1_, *ρ*_2 _∈ *e *that both use *υ*. For example, a vertex *c^h ^*is inter-reused on the branches (*Q*_3_, *P*_1_) and (*Q*_2_, *P*_3_), while a vertex *f^h ^*is intra-reused on the branch (*Q*_3_, *Q*_2_) of the tree in Figure 3. We define *br*(*e*_1_, *e*_2_) as the number of vertices inter-reused on the branches *e*_1 _and *e*_2_, and *br*(*e*) as the number of vertices intra-reused on the branch *e*. An alternative approach to measuring breakpoint intra-reuse is to define *weighted intra-reuse *of a vertex *υ *on a branch *e *as max{0, *use*(*e*, *υ*) -1} where *use*(*e*, *υ*) is the number of 2-breaks on *e *using *υ*. The weighted intra-reuse *BR*(*e *) on the branch *e *is the sum of weighted intra-reuse of all vertices. We remark that if no vertex is used more than twice on a branch *e *then *BR*(*e*) = *br*(*e*).

Given simulated data, one can compute *br*(*e*) for all branches and *br*(*e*_1_, *e*_2 _) for all pairs of branches in the phylogenetic tree. However, for real data, rearrangements along the branches are unknown, calling for alternative ways for estimating the inter- and intra-reuse.

Cycles in the breakpoint graphs provide yet another way to estimate the inter- and intra-reuse. For a branch *e *= (*P*, *Q*) of the phylogenetic tree, one can estimate *br*(*e*) by comparing the 2-break distance *d*(*P*, *Q *) and the number of breakpoints 2 · *b*(*P*, *Q*) between the genomes *P *and *Q*. This results in the lower bound *bound*(*e*) = 4 · *d*(*P*, *Q*) -2 · *b*(*P*, *Q*) for *BR*(*e*) [34] that also gives a good approximation for *br*(*e *). On the other hand, one can estimate *br*(*e*_1_, *e*_2_) as the number *bound*(*e*_1_, *e*_2_) of vertices shared between non-trivial cycles in the breakpoint graphs corresponding to the branches *e*_1 _and *e*_2 _(similar approach was used in [48] and later explored in [12,33]). Assuming that the genomes at the internal nodes of the phylogenetic tree can be reliably reconstructed [33,49-51], one can compute *bound*(*e*) and *bound*(*e*_1_, *e*_2_) for all (pairs of) branches. Below we show that these bounds accurately approximate the intra- and inter-reuse.

### Analyzing breakpoint reuse (simulated genomes)

We start from analyzing simulated data based on FBM with *n *fragile regions present in *k *genomes that evolved according to a certain phylogenetic tree (for the varying parameter *n *). We represent one of the leaf genomes as the genome with 20 random circular chromosomes and simulate hundred 2-breaks on each branch of the tree.

**Figure 4:**
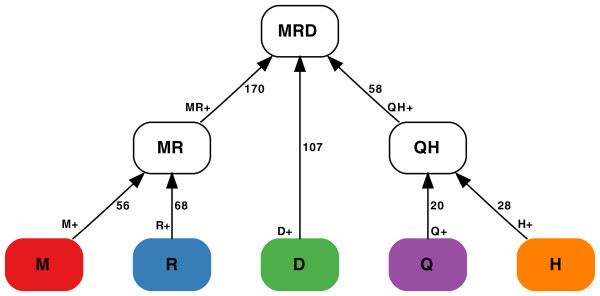
**The phylogenetic tree *T *on five genomes *M*, *R*, *D*, *Q*, and *H***. The branches of the tree are denoted as *M*+, *R*+, *D*+, *Q*+, *H*+, *MR*+, and *QH*+.

**Figure 5:**
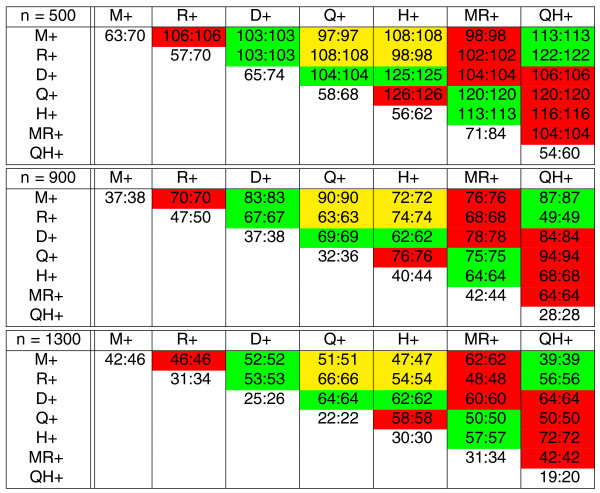
**The number of intra- and inter-reuses between seven branches of the tree in Figure 4, each of length 100, for simulated genomes with *n *fragile regions (*n *= 500, 900, 1, 300)**. The diagonal elements represent intra-reuses while the elements above diagonal represent inter-reuses. In each cell with numbers *x *: *y*, *x *represents the observed reuse while *y *represents the corresponding lower bound. The cells of the table are colored red (for adjacent branches like *M*+ and *R*+), green (for branches that are separated by a single branch like *M*+ and *D*+ separated by *MR*+), and yellow (for branches that are separated by two branches like *M*+ and *H*+ separated by *MR*+ and *QH*+).

Figure 4 represents a phylogenetic tree on five leaf genomes, denoted *M*, *R*, *D*, *Q*, *H*, and three ancestral genomes, denoted *MR*, *MRD*, *QH*. Table in Figure 5 presents the results of a single FBM simulation and illustrates that *bound*(*e*_1_, *e*_2_) provides an excellent approximation for inter-reuses *br*(*e*_1_, *e*_2 _) for all 21 pairs of branches. While *bound*(*e*) (on the diagonal of table in Figure 5) is somewhat less accurate, it also provides a reasonable approximation for *br*(*e*). We remark that *bound*(*e*_1_, *e*_2_) = *br*(*e*_1_, *e*_2_) if simulations produce the shortest rearrangement scenarios on the branches *e*_1 _and *e*_2_. Table in Figure 5 illustrates that this is mainly the case for our simulations.

Below we describe analytical approximations for the values in table in Figure 5. Since every 2-break uses four out of 2*n *vertices in the genome graph, a random 2-break uses a vertex *υ *with the probability $\frac{2}{n}$. Thus, a sequence of *t *random 2-breaks does not use a vertex *υ *with the probability ${\left(1-\frac{2}{n}\right)}^{t}\approx e^{-\frac{2t}{n}}(for\;t\ll n)$. For branches *e*_1 _and *e*_2 _with respectively *t*_1 _and *t*_2 _random 2-breaks, the probability that a particular vertex is inter-reused on *e*_1 _and *e*_2 _is approximated as $\left(1-e^{-\frac{2t_{1}}{n}})\cdot (1-e^{-\frac{2t_{2}}{n}}\right)$. Therefore, the expected number of inter-reused vertices is approximated as $2n\cdot (1-e^{-\frac{2t_{1}}{n}})\cdot (1-e^{-\frac{2t_{2}}{n}})$. Below we will compare the observed inter-reuse with the expected inter-reuse in FBM to see whether they are similar thus checking whether FBM represents a reasonable null hypothesis. We will use the term *scaled inter-reuse *to refer to the observed inter-reuse divided by the expected inter-reuse. If FBM is an adequate null hypothesis we expect the scaled inter-reuse to be close to one.

Similarly, a sequence of *t *random 2-breaks uses a vertex *υ *exactly once with the probability $t\cdot \frac{2}{n}\cdot {\left(1-\frac{2}{n}\right)}^{t-1}\approx \frac{2t}{n}e^{\frac{2(t-1)}{n}}$. Therefore, the probability of a particular vertex being intra-reused on a branch with *t *random 2-breaks is approximately $1-e^{-\frac{2t}{n}}-\frac{2t}{n}e^{\frac{2(t-1)}{n}}$, implying that the expected intra-reuse is approximately $2n\cdot \left(1-e^{-\frac{2t}{n}}-\frac{2t}{n}e^{\frac{2(t-1)}{n}}\right)$. We will use the term *scaled intra-reuse *to refer to the observed *n^e ^*intra-reuse divided by the expected intra-reuse. Table S1 in Additional file 1 shows the scaled intra- and inter-reuse for 21 pairs of branches (averaged over 100 simulations) and illustrates that they all are close to one.

**Figure 6:**
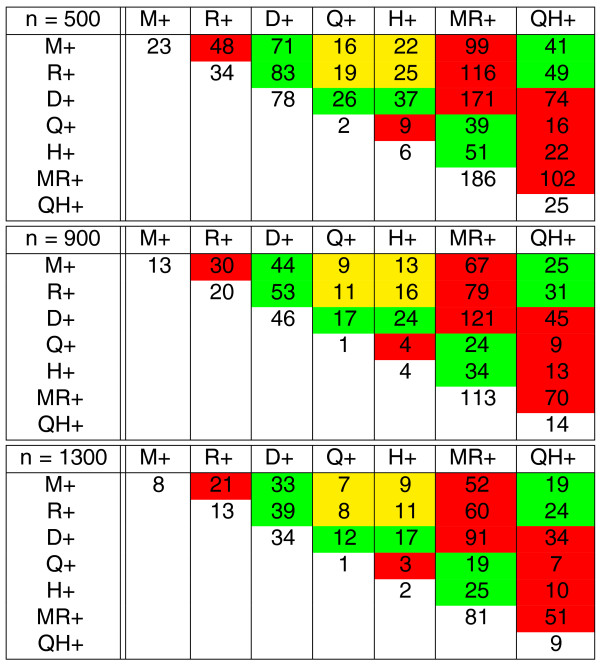
**The estimated number of intra- and inter-reuses *bound*(*e*) and *bound*(*e*_1_, *e*_2_) between seven branches with varying branch length specified in Figure 4 (data simulated according to FBM)**. The cells are colored as in Figure 5.

We now perform a similar simulation, this time varying the number of 2-breaks on the branches according to the branch lengths specified in Figure 4. Table S2 in Additional file 1 (similar to Table S1 in Additional file 1) illustrates that the lower bounds also provide accurate approximations in the case of varying branch lengths. Similar results were obtained in the case of evolutionary trees with varying topologies (data are not shown). We therefore use only lower bounds to generate table in Figure 6 rather than showing both real distances and the lower bounds as in table in Figure 5.

In the case when the branch lengths vary, we find it convenient to represent data in Table S2 in Additional file 1 in a different way (as a plot) that better illustrates variability in the scaled inter-use. We define the *distance *between branches *e*_1 _and *e*_2 _in the phylogenetic tree as the distance between their midpoints, that is, the overall length of the path, starting at *e*_1 _and ending at *e*_2_, minus $\frac{d(e_{1})+d(e_{2})}{2}$. For example, $d(M+,H+)=56+170+58+28-\frac{56+28}{2}=270$ (see Figure 4). The *x*-axis in Figure S1 in Additional file 1, 2 represents the distances between pairs of branches (21 pairs total), while *y*-axis represents the scaled inter-reuse for pairs of branches at the distance *x*.

### Surprising irregularities in breakpoint reuse in mammalian genomes

**Figure 7:**
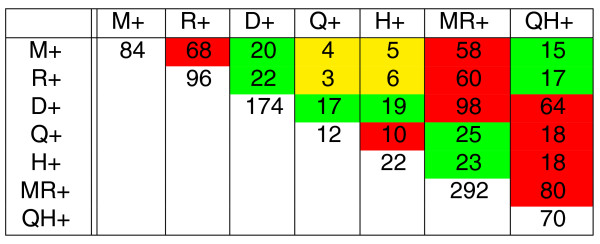
**The estimated number of intra- and inter-reuses *bound*(*e*) and *bound*(*e*_1_, *e*_2_) between seven branches of the phylogenetic tree in Figure 4 of five mammalian genomes (real data)**. The cells are colored as in Figure 5.

The branch lengths shown in Figure 4 actually represent the approximate numbers of rearrangements on the branches of the phylogenetic tree for *M*ouse, *R*at, *D*og, maca*Q*ue, and *H*uman genomes (represented in the alphabet of 433 'large' synteny blocks exceeding 500, 000 nucleotides in human genome [50]). For the mammalian genomes, *M*, *R*, *D*, *Q*, and *H*, we first used MGRA [50] to reconstruct genomes of their common ancestors (denoted *MR*, *MRD*, and *QH *in Figure 4) and further estimated the breakpoint inter-reuse between pairs of branches of the phylogenetic tree. The resulting table in Figure 7 reveals some striking differences from the simulated data (Figure 6) that follow a peculiar pattern: the larger is the distance between two branches, the smaller is the amount of inter-reuse between them (in contrast to RBM/FBM where the amount of inter-reuse does not depend on the distance between branches). The statement above is imprecise since we have not described yet how to compare the amount of inter-reuse for different branches at various distances. However, we can already illustrate this phenomenon by considering branches of similar length that presumably influence the inter-reuse in a similar way (see below).

**Figure 8:**
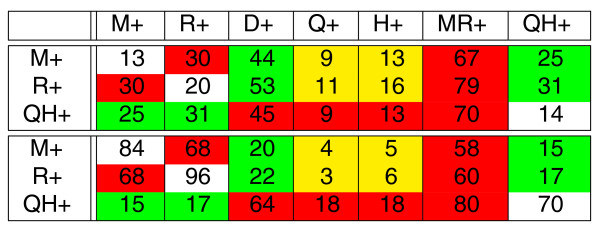
**Subtables of Figure 6 for *n *= 900 (top part) and Figure 7 (bottom part) featuring branches *M*+, *R*+, and *QH*+ as one element of the pair**. The cells are colored as in Figure 5.

We notice that branches *M*+, *R*+, and *QH*+ have similar lengths (varying from 56 to 68 rearrangements) and construct subtables of Figure 6 (for *n *= 900) and Figure 7 with only three rows corresponding to these branches (Figure 8). Since the lengths of branches *M*+, *R*+, and *QH*+ are similar, FBM implies that the elements belonging to the same columns in table in Figure 8 should be similar. This is indeed the case for simulated data (small variations within each column) but not the case for real data. In fact, maximal elements in each column for real data exceed other elements by a factor of three to five (with an exception of the *MR*+ column). Moreover, the peculiar pattern associated with these maximal elements (maximal elements correspond to red cells) suggests that this effect is unlikely to be caused by random variations in breakpoint reuses. We remind the reader that red cells correspond to pairs of adjacent branches in the evolutionary tree suggesting that breakpoint reuse is maximal between close branches and is reducing with evolutionary time. A similar pattern is observed for the other pairs of branches of similar length: adjacent branches feature much higher inter-reuse than distant branches. We also remark that the most distant pairs of branches (*H*+ and *M*+, *H*+ and *R*+, *Q*+ and *M*+, *Q*+ and *R*+ in the yellow cells) feature the lowest inter-reuse. The only branch that shows relatively similar inter-reuse (varying from 58 to 80) with the branches *M*+, *R*+, and *QH*+ is the branch *MR*+ which is adjacent to each of these branches.

Below we modify FBM to come up with a new model of chromosome evolution, explaining the surprising irregularities in the inter-reuse across mammalian genomes.

### Turnover fragile breakage model: birth and death of fragile regions

We start with a simulation of 100 rearrangements on every branch of the tree in Figure 4. However, instead of assuming that fragile regions are fixed, we assume that after every rearrangement *x *fragile regions 'die' and *x *fragile regions are 'born' (keeping a constant number of fragile regions throughout the simulation). We assume that the genome has *m *potentially 'breakable' sites but only *n *of them are currently fragile (*n *≤ *m*) (the remaining *n - m *sites are currently solid). The dying regions are randomly selected from *n *currently fragile regions, while the newly born regions are randomly selected from *m - n *solid regions. The simplest TFBM with a fixed rate of the 'birth and death' process is defined by the parameters *m*, *n*, and *turnover *rate *x*. FBM is a particular case of TFBM corresponding to *x *= 0 and *n *<*m*, while RBM is a particular case of TFBM corresponding to *x *= 0 and *n *= *m*. While this over-simplistic model with a fixed turnover rate may not adequately describe the real rearrangement process, it allows one to analyze the general trends and to compare them to the trends observed in real data. We further remark that the goal of this paper is to develop a test for distinguishing between TFBM and FBM/RBM rather than a test for distinguishing between FBM and RBM. Thus, our simulations do not distinguish between FBM (*x *= 0 and *n *<*m*) and RBM (*x *= 0 and *n *= *m*) since they do not affect *m - n *inactive breakpoints in FBM. To distinguish FBM from RBM, one has to analyze the long cycles in the breakpoint graph and the distribution of synteny block sizes (see [3,8]).

**Figure 9:**
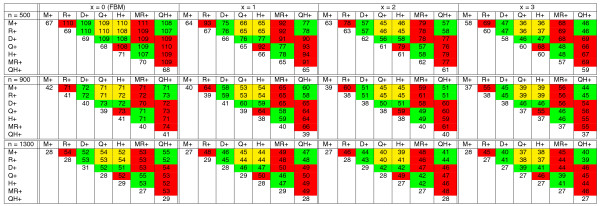
**The breakpoint intra- and inter-reuse (averaged over 100 simulations) for five simulated genomes *M*, *R*, *D*, *Q*, *H *under TFBM model with *m *= 2, 000 synteny blocks, *n *fragile regions, the turnover rate *x*, and the evolutionary tree shown in Figure 4 with the length of each branch equal 100**. The cells are colored as in Figure 5.

The leftmost subtable of Figure 9 with *x *= 0 represents an equivalent of table in Figure 5 for FBM and reveals that the inter-reuse is roughly the same on all pairs of branches (approximately 110 for *n *= 500, approximately 70 for *n *= 900, approximately 50 for *n *= 1, 300). The right subtables of Figure 9 represent equivalents of the leftmost subtable for TFBM with the turnover rate *x *= 1, 2, 3 and reveal that the inter-reuse in yellow cells is lower than in green cells, while the inter-reuse in green cells is lower than in red cells.

**Figure 10:**
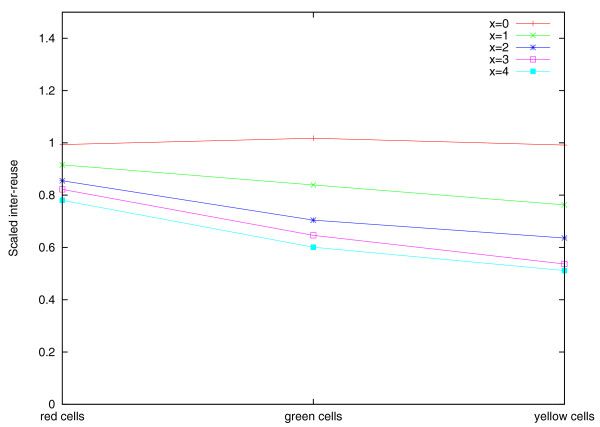
**The scaled inter-reuse for five simulated genomes *M*, *R*, *D*, *Q*, *H *on *m *= 2,000 synteny blocks, *n *= 900 fragile regions, and the turnover rate *x *varying from zero to four with the phylogenetic tree and branch lengths shown in Figure 4**. The simulations follow FBM (*x *= 0) and TFBM (*x *varies from one to four). The plot shows the scaled inter-reuse for only three reference points (corresponding to red, green, and yellow cells) that are somewhat arbitrarily connected by straight segments for better visualization.

Figure 10 shows the scaled inter-reuse averaged over yellow, green, and red cells that reveals a different behavior between FBM and TFBM. Indeed, while the scaled inter-reuse is close to 1 for all pairs of branches in the case of FBM, it varies in the case of TFBM. For example, for *n *= 900, *m *= 2, 000, and *x *= 3, the inter-reuse in yellow cells is approximately 40, in green cells is approximately 45, and in red cells is approximately 56. Table S3 in Additional file 1 presents the differences in the inter-reuse between red, green, and yellow cells as a function of *m *and *x *(for *n *= 900). In Methods we describe a formula for estimating the breakpoint inter-reuse in the case of TFBM that accurately approximates the values shown in Figure 10.

Table S3 in Additional file 1 demonstrates that the distribution of inter-reuses among green, red, and yellow cells differs between FBM and TFBM. We argue that this distribution (for example, the slope of the curve in Figure 10) represents yet another test to confirm or reject FBM/TFBM. However, while it is clear how to apply this test to the simulated data (with known rearrangements), it remains unclear how to compute it for real data when the ancestral genomes (as well as the parameters of the model) are unknown. While the ancestral genomes can be reliably approximated using the algorithms for ancestral genome reconstruction [33,49-51], estimating the number of fragile regions remains an open problem (see [3]). Below we develop a new test (that does not require knowledge of the number of the fragile regions *n *) and demonstrate that FBM does not pass this test while TFBM does, explaining the surprisingly low inter-reuse in mammalian genomes.

### Multispecies breakpoint reuse test

Given a phylogenetic tree describing a rearrangement scenario, we define the multispecies breakpoint reuse on this tree as follows. For two rearrangements *ρ*_1 _and *ρ*_2 _in the scenario, we define the distance *d*(*ρ*_1_, *ρ*_2_) as the number of rearrangements in the scenario between *ρ*_1 _and *ρ*_2 _plus one. For example, the distance between 2-breaks *r*_4 _and *r*_6 _in the tree in Figure 3 is four. We define the (actual) multispecies breakpoint reuse as a function

$$R(\ell )=\frac{\sum_{\rho_{1},\rho_{2}\;:\;d(\rho_{1},\rho_{2})=\ell }br(\rho_{1},\rho_{2})}{\sum_{\rho_{1,}\rho_{2}\;:\;d(\rho_{1},\rho_{2})=\ell }1}$$

that represents the total breakpoint reuse between pairs of rearrangements *ρ*_1_, *ρ*_2 _at the distance *l *divided by the number of such pairs. Here *br*(*ρ*_1_, *ρ*_2_) stands for the number of vertices used by both 2-breaks *ρ*_1 _and *ρ*_2_.

Since the rearrangements on branches of the phylogenetic tree are unknown, we use the following sampling procedure to approximate *R*(*l*). Given genomes *P *and *Q*, we sample various shortest rearrangement scenarios between these genomes by generating random 2-break transformations of *P *into *Q*. To generate a random transformation we first randomly select a non-trivial cycle *C *in the breakpoint graph *G*(*P*, *Q*) with the probability proportional to |*C*|/ = 2 - 1, that is, the number of 2-breaks required to transform such a cycle into a collection of trivial cycles (|*C*| stands for the length of *C*). Then we uniformly randomly select a 2-break *ρ *from the set of all ${(}_{2}^{{}^{|\text{C}|/2}})=\frac{\left|\text{C}|(|\text{C}|-2\right)}{8}$ 2-breaks that splits the selected cycle *C *into 2 8 two and thus by Theorem 1 decreases the distance between *P *and *Q *by one (that is, *d*(*ρ P*, *Q*) = *d*(*P*, *Q*) -1). We continue selecting non-trivial cycles and 2-breaks in an iterative fashion for genomes *ρ *· *P *and *Q *and so on until *P *is transformed into *Q*.

The described sampling can be performed for every branch *e *= (*P*, *Q*) of the phylogenetic tree, essentially partitioning *e *into *length*(*e*) = *d*(*P*, *Q*) sub-branches, each featuring a single 2-break. The resulting tree will have ∑_*e *_*length*(*e*) sub-branches, where the sum is taken over all branches *e*.

For each pair of sub-branches, we compute the number of reused vertices across them and accumulate these numbers according to the distance between these sub-branches in the tree. The *empirical multispecies breakpoint reuse *(the average reuse between all sub-branches at the distance *l*) is defined as the actual multispecies breakpoint reuse in a sampled rearrangement scenario. Figure S2 in Additional file 1 represents this function for five simulated genomes on *m *= 2, 000 synteny blocks, *n *= 900 fragile regions, and the turnover rate *x *varying from zero to four, with the same phylogenetic tree and distances between the genomes (averaged over 100 random samplings, while individual samplings produce varying results, we found that the variance of the *R*(*l*) estimates across various samplings is rather small). Figure S3 in Additional file 1 demonstrates that our sampling procedure, while imperfect, accurately estimates the theoretical *R*(*l*) curve (see [52] for other approaches to sampling rearrangement scenarios). Similar tests on phylogenetic trees with varying topologies demonstrated a good fit between actual, empirical, and theoretical *R*(*l*) curves (data are not shown).

**Figure 11:**
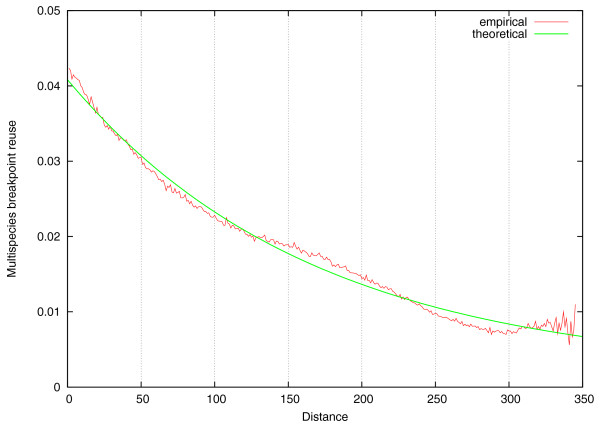
**Empirical and theoretical curves representing the number of reuses *R*(*l*) as a function of distance *l *between pairs of sub-branches of the tree in Figure 4 of the five mammalian genomes (ancestral genomes were computed using MGRA **[50]**)**. The empirical curve is averaged over 1, 000 random samplings of shortest rearrangement scenarios, while the theoretical curve represents the best fit with parameters *n *≈ 196, *x *≈ 1:12, and *m *≈ 4, 017 (see Methods).

For the five mammalian genomes, the plot of *R*(*l*) is shown in Figure 11. From this empirical curve we estimated the parameters *n *≈ 196, *x *≈ 1:12, and *m *≈ 4, 017 (see Methods) and displayed the corresponding theoretical curve. We remark that the estimated parameter *n *in TFBM is expected to be larger than the observed number of synteny blocks (since not all potentially breakable regions were broken in a given evolutionary scenario). Figure S4 in Additional file 1 represents an analog of Figure 11 for the same genomes in higher resolution and illustrates that all three parameters *n*, *x*, and *m *depend on the data resolution.

We argue that the empirical multispecies breakpoint reuse curve *R*(*l*) complements the 'exponential length distribution' [2] and 'pairwise breakpoint reuse' [3] tests as the third criterion to accept/reject RBM, FBM, and now TFBM. One can use the parameters *n *and *x *(estimated from empirical *R*(*l*) curve) to evaluate the extent of the 'birth and death' process and to explain why Ma *et al. *[33] found so few shared breakpoints between different mammalian lineages. In practice, the 'multispecies breakpoint reuse test' can be applied in the same way as the Nadeau-Taylor 'exponential length distribution test' was applied in numerous papers. The Nadeau-Taylor test typically amounted to constructing a histogram of synteny blocks and evaluating (often visually) whether it fits the exponential distribution. Similarly, the 'multispecies breakpoint reuse test' amounts to constructing *R*(*l*) curve and evaluating whether it significantly deviates from a horizontal line suggested by RBM and FBM. The estimated parameters of the TFBM model (see Methods) can be used to quantify the extent of these deviations.

TFBM also raises an intriguing question of what triggers the birth and death of fragile regions. As demonstrated by Zhao and Bourque [38], the disproportionately large number of rearrangements in primate lineages are flanked by MSDs. TFBM is consistent with the Zhao-Bourque hypothesis that rearrangements are triggered by MSDs since MSDs are also subject to the 'birth and death' process. Indeed, after a segmental duplication the pair of matching segments becomes subjected to random mutations and the similarity between these segments dissolves with time (a pair of segmental duplications 'disappears' after approximately 40 million years of evolution if one adopts the parameters for defining segmental duplications from [53]).

The mosaic structure of segmental duplications [53] provides an additional explanation of how MSDs may promote breakpoint re-uses and generate long cycles typical for the breakpoint graphs of mammalian genomes. The future studies of the correlation between fragile regions and MSDs in the human genome will benefit from the algorithms for precise detection of rearrangement breakpoints [54] and will be described elsewhere.

### Fragile regions in the human genome

Imagine the following gedanken experiment: 25 million years ago (time of the human-macaque split) a scientist sequences the genome of the human-macaque ancestor (*QH*) and attempts to predict the sites of (future) rearrangements in the (future) human genome. The only other information the scientist has is the mouse, rat, and dog genomes. While RBM offers no clues on how to make such a prediction, FBM suggests that the scientist should use the breakpoints between one of the available genomes and *QH *as a proxy for fragile regions. For example, there are 552 breakpoints between the mouse genome (*M*) and *QH *and 34 of them were actually used in the human lineage, resulting in only 34 = 552 ≈ 6% accuracy in predicting future human breakpoints (we use synteny blocks larger than 500 K from [50]).

TFBM suggests that the scientist should rather use the *closest *genome to *QH *to better predict the human breakpoints. That can be achieved by first reconstructing the common ancestor (*MRD*) of mouse, rat, dog, and human-macaque ancestor and then using the breakpoints between *MRD *and *QH *as a proxy for the sites of rearrangements in the human lineage. 18 out 162 breakpoints between *MRD *and *QH *were used in the human lineage, resulting in 18 = 162 ≈ 11% accurate prediction of human breakpoints, nearly doubling the accuracy of predictions from distant genomes.

Now imagine that the scientist somehow gained access to the extant macaque genome. There are 68 breakpoints between *Q *and *QH *and 10 of them were used in the human lineage, resulting in 10 = 68 ≈ 16% accurate prediction of human breakpoints, again improving the accuracy of predictions. These estimates indicate that TFBM can be used to improve the prediction accuracy of *future *rearrangements in various lineages and demonstrate that the sites of *recent *rearrangements in the human and other primate lineages represent the best guess for the currently active fragile regions in the human genome.

**Figure 12:**
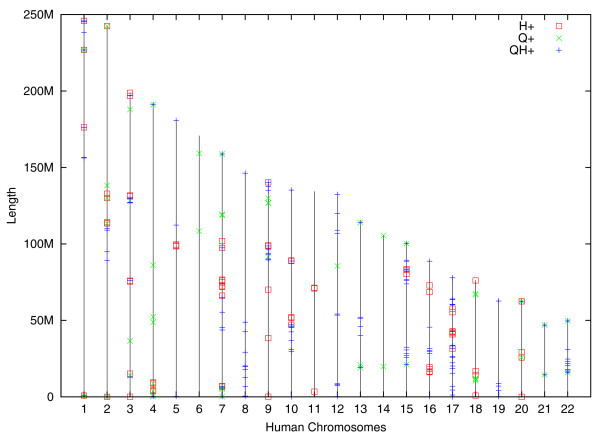
**Positions of regions broken on the evolutionary path from the rodent-primate-carnivore ancestor (that is, on *H*+, *Q*+, and *QH*+ branches) projected to the human chromosomes**.

We therefore focus on the incident branches *H*+, *Q*+, and *QH*+ and construct the breakpoint graphs *G*(*H*, *QH*), *G*(*Q*, *QH*), and *G*(*QH*, *MRD*). Figure S5 in Additional file 1 superimposes these three graphs and (together with Table S4 in Additional file 1) illustrates breakpoints that were inter-reused on the branches *H*+, *Q*+, and *QH*+. Figure 12 shows the positions of these recently affected breakpoints (projected to the human genome) that, according to TFBM, represent the best proxy for the currently active fragile regions in the human genome. Various ongoing primate genome sequencing projects will soon result in an even better estimate for the fragile regions in the human genome.

## Conclusions

Since every species on Earth (including *Homo sapiens*) may speciate into multiple new species, one can ask a question: 'How will the human genome evolve in the *next *million years?' TFBM suggests the putative sites of *future *rearrangements in the human genome. The answer to the question 'Where are the (future) fragile regions in the human genome?' may be surprisingly simple: they are likely to be among the breakpoint regions that were used in various primate lineages.

Nadeau and Taylor [2] proposed RBM based on a single observation: the exponential distribution of the human-mouse synteny block sizes. There is no doubt that jumping to this conclusion was not fully justified: there are many other models (for example, FBM) that lead to the same exponential distribution of the 'visible' synteny block sizes. Currently, there is no single piece of evidence that would allow one to claim that RBM is correct and FBM is not.

While Pevzner and Tesler [3] revealed large breakpoint reuse (supporting FBM and contradicting RBM), Ma *et al. *[33] revealed low breakpoint inter-reuse (contradicting FBM). This discovery calls for yet another generalization of FBM. The proposed TFBM model not only passes both 'exponential length distribution' test (motivation for RBM) and 'pairwise breakpoint reuse' test (motivation for FBM) but also explains the puzzling discovery of limited breakpoint inter-reuse in [33]. We therefore argue that TFBM is a more accurate model of chromosome evolution, allowing one to approximate the currently active fragile regions in the human genome.

Needless to say, TFBM, similarly to RBM and FBM (or various models of point mutations, for example, Jukes-Cantor model), is a simplistic model of chromosome evolution that is only an approximation of the real evolutionary process. Moreover, in the current paper we considered TFBM only for the case of 2-breaks and did not include other rearrangements such as transpositions. However, it is fair to assume that transpositions are as likely to happen on incident branches as on distant branches, implying that they cannot possibly cause the reduced breakpoint inter-reuse on distant branches. In addition to limitations of TFBM as a model, there exists a concern whether computation of empirical multispecies breakpoint reuse (that requires reconstruction of ancestral genomes) may be affected by errors in reconstruction of ancestral genomes. While various tools for ancestral genome reconstruction (such as MGRA [50] and inferCARs [33]) were shown to be quite accurate (in particular, they produce nearly identical results while using very different algorithms), it is a challenging open problem to evaluate the multispecies breakpoint reuse without explicitly computing ancestral genomes.

The key point of this paper is the birth and death process of fragile regions rather than a specific model aimed at estimating the hidden parameters of this process. TFBM is merely an initial and over-simplistic attempt to estimate these parameters. The parameters predicted by TFBM (for example, the number of active fragile regions) are currently difficult to superimpose with scarce information about rearrangements in only seven reliably completed mammalian genomes, not unlike the parameters of RBM derived in 1984 when no high-resolution comparative mammalian genomic architectures were available. However, similarly to comparative mapping efforts in early 1990 s that confirmed the Nadeau-Taylor estimates, we believe that imminent sequencing of over 400 primate species will soon provide the detailed information about chromosomal fragility in human genome and will allow one to verify the TFBM parameters.

Similarly to the discovery of breakpoint reuse in 2003 [3], there is currently only indirect evidence supporting the birth and death of fragile regions in chromosome evolution. However, we hope that, similarly to FBM (that led to many follow-up studies supporting the existence of fragile regions), TFBM will trigger further investigations of the fragile regions longevity.

## Materials and methods

### Computing multispecies breakpoint reuse in the TFBM model

Let *Fragile *and *Solid *be the sets of *n *initial fragile regions and *m - n *initial solid regions respectively. In TFBM, the sets *Fragile *and *Solid *change in accordance with the turnover rate *x*, that is, after every 2-break *x *fragile regions (corresponding to 2*x *vertices in the breakpoint graph) from *Fragile *are moved to *Solid *and vice versa.

For a vertex in the set *Fragile*, we evaluate the probability *P*(*l*) that this vertex still belongs to *Fragile *after *l *2-breaks. After every 2-break, a vertex from *Fragile *moves to *Solid *with the probability $\frac{x}{n}$, while a vertex from *Solid *moves to *Fragile *with the probability $\frac{x}{m-n}$. Therefore,

P(ℓ+1)=P(ℓ)⋅(1−xn)+(1−P(ℓ))⋅xm−n=(1−xmn(m−n))⋅P(ℓ)+xm−n.

Solution to this recurrence with the initial condition *P*(0) = 1 is $P(\ell )=\frac{m-n}{m}{\left(1-\frac{xm}{n(m-n)}\right)}^{\ell }+\frac{n}{m}$. We now compute the expected reuse between 2-breaks *ρ*_1 _and *ρ*_2 _separated by *l *other 2-breaks. Since every 2-break uses 4 vertices, the probability that it uses a particular vertex in *Fragile *is $\frac{2}{n}$. Since the 2-break used 4 vertices, the expected reuse between *ρ*_1 _and *ρ*_2 _is:

$$R(\ell )=4\cdot \frac{2}{n}\cdot P(\ell )=\frac{8\cdot (m-n)}{n\cdot m}{\left(1-\frac{xm}{n(m-n)}\right)}^{\ell }+\frac{8}{m}.$$

Figure S6 in Additional file 1 demonstrates that this formula fits simulated data well, thus opening a possibility to determine the parameters *m*, *n*, and *x *for given real genomes.

We remark that if $\frac{xml}{n(m-n)}\ll 1$ is approximated by a line $\frac{8\cdot (m-n)}{n\cdot m}\left(1-\frac{xm}{n(m-n)}\ell \right)+\frac{8}{m}=\frac{8}{n}-\frac{8x}{n^{2}}\ell$ that does not depend on *m*.

### The difference between empirical and theoretical estimates for *R*(*l*)

Figure S3 in Additional file 1 illustrates the results of simulating of 400 2-breaks according to TFBM with parameters *m *= 2, 000, *n *= 900, *x *= 1. As expected, the theoretical curve and the curve derived from simulated data (without sampling of various rearrangement scenarios) are nearly identical. We now assume that only five out of 401 simulated genomes are available (after 0, 100, 200, 300, and 400 rearrangements) and use sampling of rearrangement scenarios to compute the empirical *R*(*l*) (Figure S3 in Additional file 1). One can see that empirical *R*(*l*) differs from the theoretical *R*(*l*), particularly for small'. To understand why the empirical curve (obtained via sampling of rearrangement scenarios) differs from the theoretical curve, one has to realize that the multispecies breakpoint reuse test requires *multiple *genome to reveal the 'birth and death' of fragile regions. Indeed, it is impossible to detect this process from only two genomes: for example, sampling of rearrangement scenarios on a single branch (simulated with TFBM with parameters described above) produces a nearly horizontal curve *R*(*l*) ≈ 0.0083 with TFBM signal lost. The green curve follows the same horizontal trend for small *l *(for example *l *< 100) that typically represent pairs of 2-breaks on the *same *branch. However, for distances larger than the shortest branches, the theoretical curve approximates the empirical *R*(*l*) curve well. The reason this 'horizontal trend' is not seen in Figure 11 most likely explained by the fact that *H*+ and *Q*+ branches in the corresponding phylogenetic tree are rather short thus masking this effect.

## Abbreviations

FBM: fragile breakage model: MSDs: matching segmental duplications: RBM: random breakage model: TFBM: turnover fragile breakage model.

## Authors' contributions

Both authors participated in data analysis and writing the manuscript. MA also performed the simulations and prepared illustrations. Both authors read and approved the final manuscript.

## Supplementary Material

Additional file 1 **Supplementary tables and figures**. Additional file 1 contains supplementary Tables S1, S2, S3, S4 and Figures S1, S2, S3, S4, S5, S6. Click here for file
