# Supplementary material for: Comparative genomics reveals birth and death of fragile regions in mammalian evolution
Source: Genome Biol. 2010 Nov 30;11(11):R117. doi: 10.1186/gb-2010-11-11-r117 (PMC3156956; doi:10.1186/gb-2010-11-11-r117)
Supplement: Additional file 1 — Supplementary tables and figures. Additional file 1 contains supplementary Tables S1, S2, S3, S4 and Figures S1, S2, S3, S4, S5, S6. [file gb-2010-11-11-r117-S1.pdf]

Supplementary Tables and Figures

n = 500	M+	R+	D+	Q+	H+	MR+	QH+
M+	1.02:1.16	1.01:1.01	0.99:0.99	0.99:0.99	1:1	1:1	1.01:1.01
R+		1.02:1.16	1:1	1:1	1.01:1.01	1.01:1.01	1:1
D+			1.03:1.18	1:1	0.99:0.99	1:1	0.99:0.99
Q+				1.02:1.16	1:1	1:1	1:1
H+					1.03:1.17	1.01:1.01	1.01:1.01
MR+						1.03:1.17	1.01:1.01
QH+							0.99:1.13

n = 900	M+	R+	D+	Q+	H+	MR+	QH+
M+	1.05:1.13	0.99:0.99	1.01:1.01	1:1	1.01:1.01	1:1	1:1
R+		1.02:1.1	0.99:0.99	1.01:1.01	0.99:0.99	0.98:0.98	1:1
D+			1.01:1.08	1:1	1:1	1.01:1.01	0.98:0.98
Q+				1.01:1.09	1.02:1.02	0.98:0.98	0.99:0.99
H+					1.02:1.09	1.02:1.02	1.01:1.01
MR+						1.03:1.11	1:1
QH+							1.01:1.09

n = 1300	M+	R+	D+	Q+	H+	MR+	QH+
M+	1.01:1.06	0.99:0.99	0.99:0.99	0.99:0.99	0.98:0.98	1.01:1.01	1.03:1.03
R+		1.02:1.07	0.97:0.97	1.01:1.01	1:1	0.99:0.99	0.99:0.99
D+			1.02:1.07	1.01:1.01	0.98:0.98	0.99:0.99	0.98:0.98
Q+				1.04:1.09	1.02:1.02	0.98:0.98	1.01:1.01
H+					0.97:1.02	0.99:0.99	1.01:1.01
MR+						1.04:1.09	1:1
QH+							0.98:1.03

Table S1: The scaled breakpoint intra- and inter-reuse (averaged over 100 simulations) for five simulated genomes M, R, D, Q, H with n fragile regions ($n = 500, 900$ and 1300) and the evolutionary tree shown in Fig. 4 where each branch is of length 100. In each cell with numbers $x : y$, x represent the scaled breakpoint reuse while y represents the scaled lower bound.

n = 500	M+	R+	D+	Q+	H+	MR+	QH+
M+	0.99	0.99	1.02	1	0.98	1.01	1
R+		1.03	1.01	1.03	1	1.01	0.99
D+			0.99	0.98	1.01	1.02	0.99
Q+				1.06	1.03	1.02	0.96
H+					1.13	1.03	1.01
MR+						1	1.01
QH+							1.03

n = 900	M+	R+	D+	Q+	H+	MR+	QH+
M+	0.95	0.98	1	0.98	1.03	1.02	1.01
R+		1.01	0.98	0.99	1	1.02	0.99
D+			0.99	0.97	1	1.02	1.01
Q+				1.05	1.07	1.01	0.99
H+					1.12	1	0.98
MR+						1.01	1.02
QH+							0.97

n = 1300	M+	R+	D+	Q+	H+	MR+	QH+
M+	1.04	1	0.98	0.99	1	0.99	0.98
R+		1.01	1	1.04	1	1.01	1.02
D+			1.03	0.94	1	0.99	0.93
Q+				1.19	1.04	1	0.92
H+					1	0.99	1.04
MR+						1.01	1.01
QH+							1

Table S2: The scaled breakpoint intra- and inter-reuse (averaged over 100 simulations) for five simulated genomes M, R, D, Q, H with n fragile regions and the evolutionary tree shown in Fig. 4 with the specified lengths of branches.

m	$x = 0$	$x = 1$	$x = 2$	$x = 3$	$x = 4$
1400	72:73:72	66:62:56	60:56:50	56:52:49	55:50:49
1600	71:71:72	63:61:56	60:52:49	58:50:44	54:46:44
1800	72:70:70	65:59:52	59:49:43	55:47:40	52:44:39
2000	71:74:71	65:59:55	59:51:45	54:45:40	51:40:36
2200	72:71:72	64:58:54	59:50:45	56:45:37	51:40:33
2400	72:71:72	65:58:56	59:50:43	56:43:41	49:39:35
2600	72:71:72	63:58:52	58:49:41	54:43:38	50:38:31
2800	72:72:71	66:61:52	57:49:41	55:41:35	52:37:29
3000	71:73:72	65:60:51	59:49:43	55:42:33	50:37:30

Table S3: The breakpoint inter-reuses (averaged over 10 simulations) for five simulated genomes M, R, D, Q, H (with the phylogenetic tree shown in Fig. 4 and the length of each branch equal 100) under TFBM model with $n = 900$ fragile regions, varying the number of synteny blocks m from 1400 to 3000 and the turnover rate x from 0 to 4. Three numbers in each cell represent the averaged breakpoint inter-reuse between pairs of adjacent branches (red cells), branches at the distance 1 (green cells), and branches at the distance 2 (yellow cells), respectively.

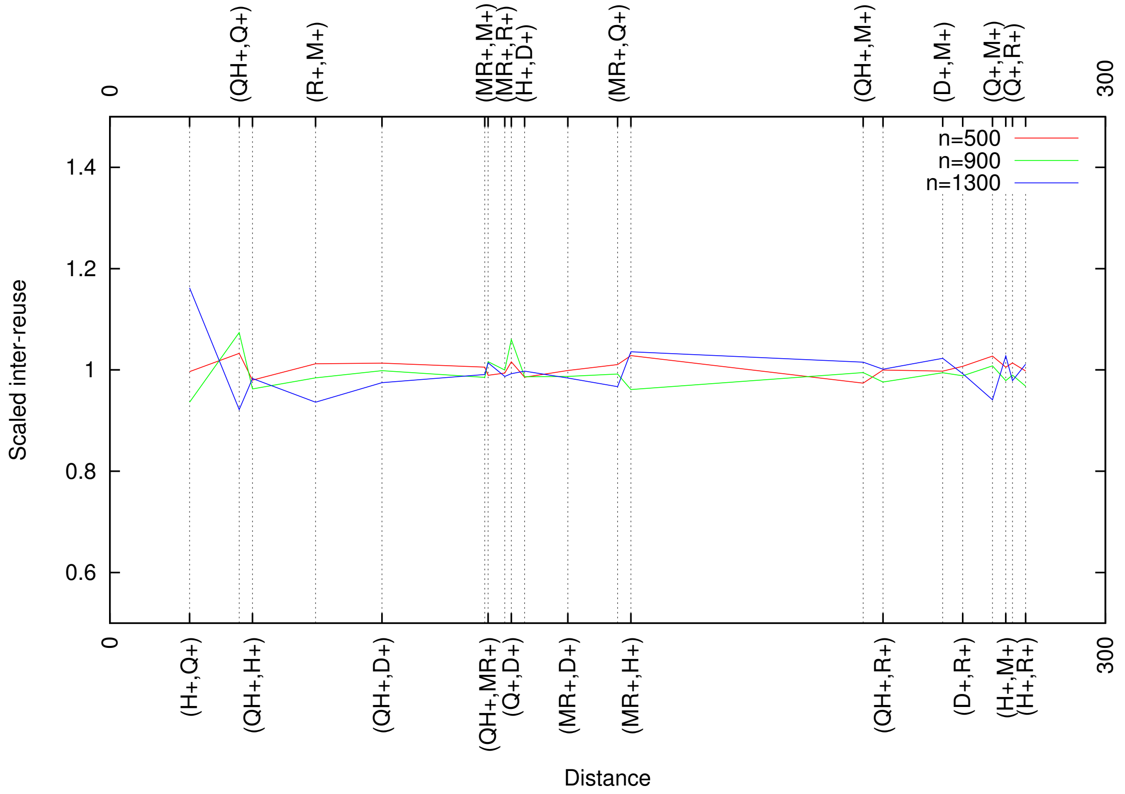

Figure S1: The scaled inter-reuse for five simulated genomes M, R, D, Q, H (averaged over 100 simulations) on n fragile regions (for $n = 500$, 900, and 1300) with the evolutionary tree and branch lengths shown in Fig. 4.

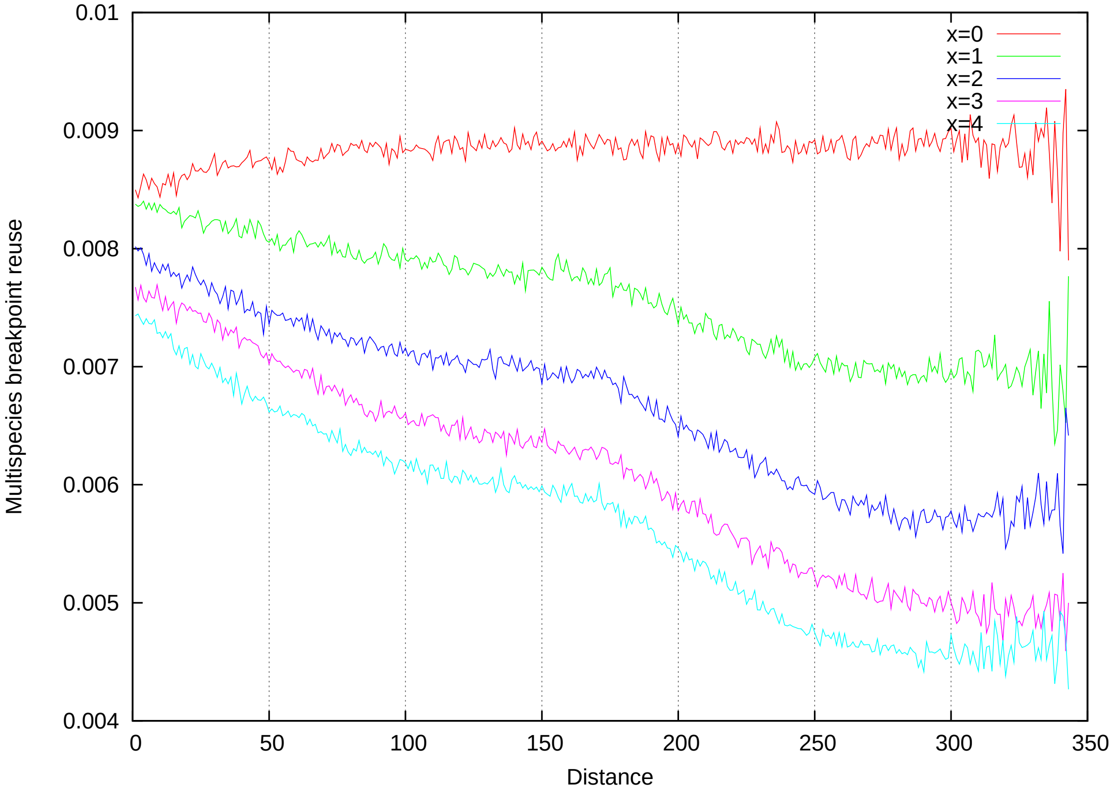

Figure S2: The multispecies breakpoint reuse $R(\ell)$ for the tree in Fig. 4 of five simulated genomes M, R, D, Q, H and their ancestral genomes MR, QH, MRD (averaged over 100 simulations and 100 random samplings of shortest rearrangement scenarios). The turnover rate x varies from 0 to 4 in these simulations ($n = 900, m = 2000$).

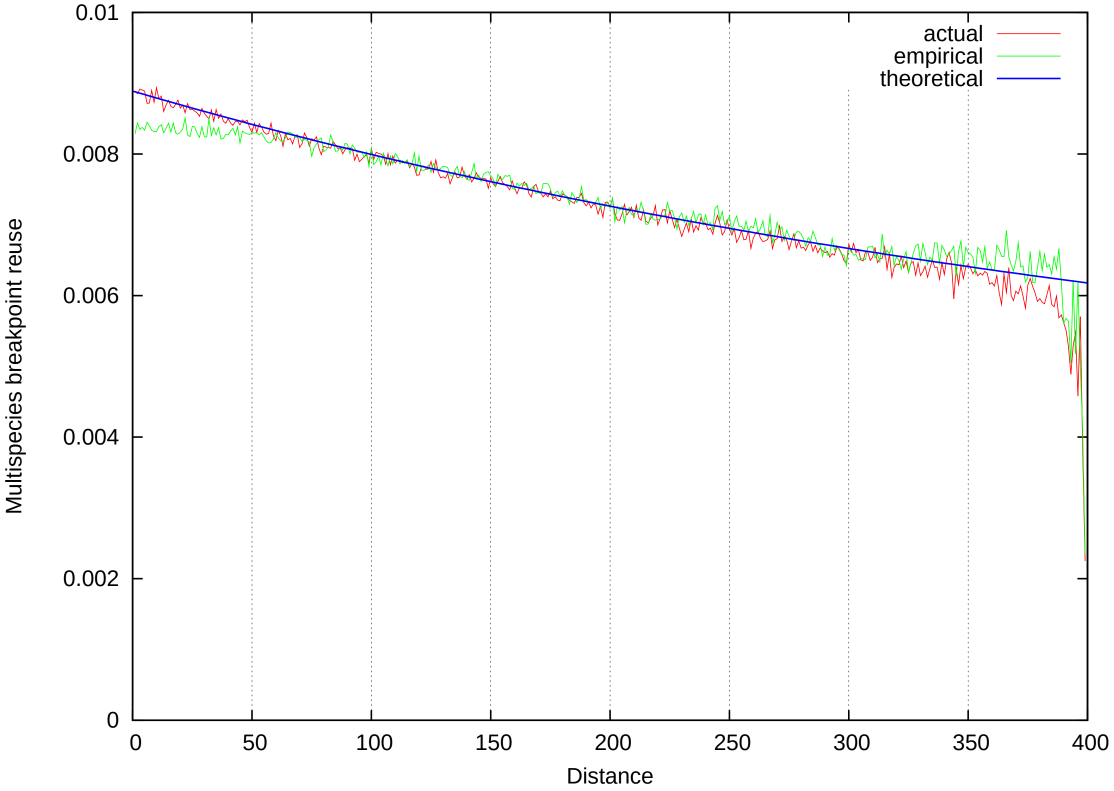

Figure S3: The actual, empirical, and theoretical multispecies breakpoint reuse curves $R(\ell)$ for a simulated phylogenetic tree formed by a path of four branches with 100 rearrangements each ($x = 1$, $n = 900$, $m = 2000$).

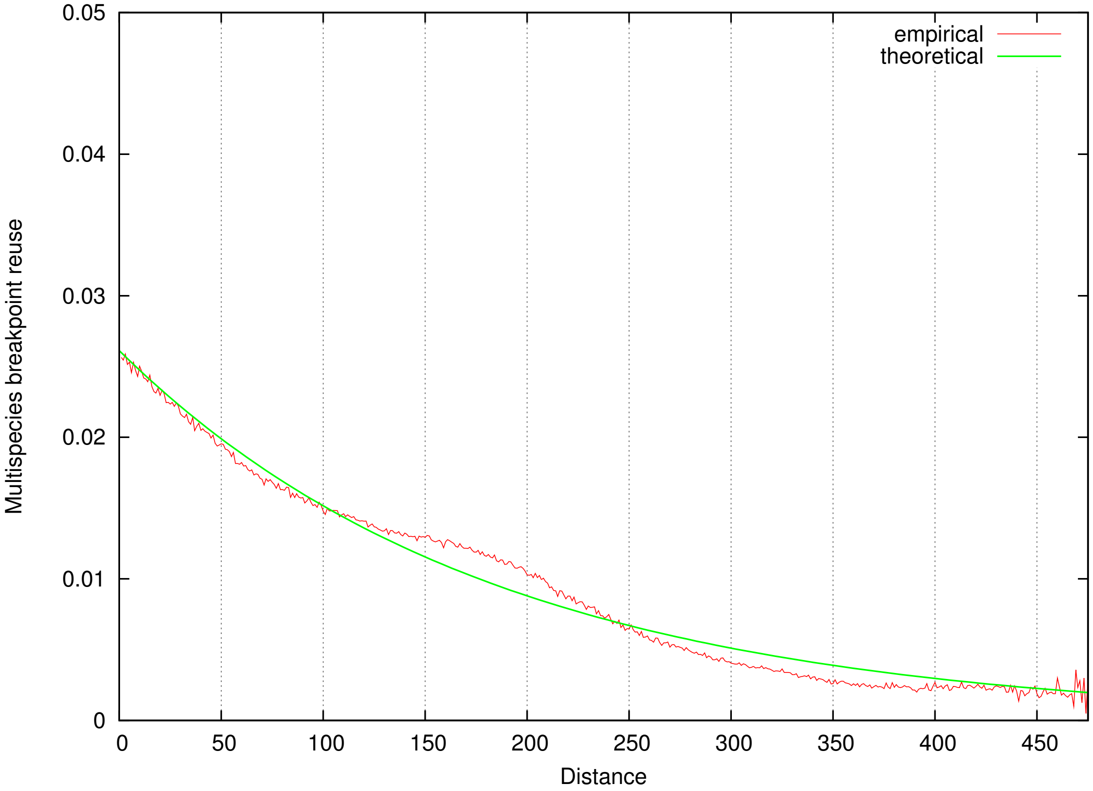

Figure S4: Empirical and theoretical curves representing the number of reuses $R(\ell)$ as a function of distance ℓ between pairs of sub-branches of the five mammalian genomes (as in Fig. 11) on 723 synteny blocks larger than $200K$. The empirical curve is averaged over 1000 random samplings of shortest rearrangement scenarios, while the theoretical curve represents the best fit with parameters $n \approx 306$, $x \approx 1.66$, and $m \approx 7.2 \cdot 10^8$.

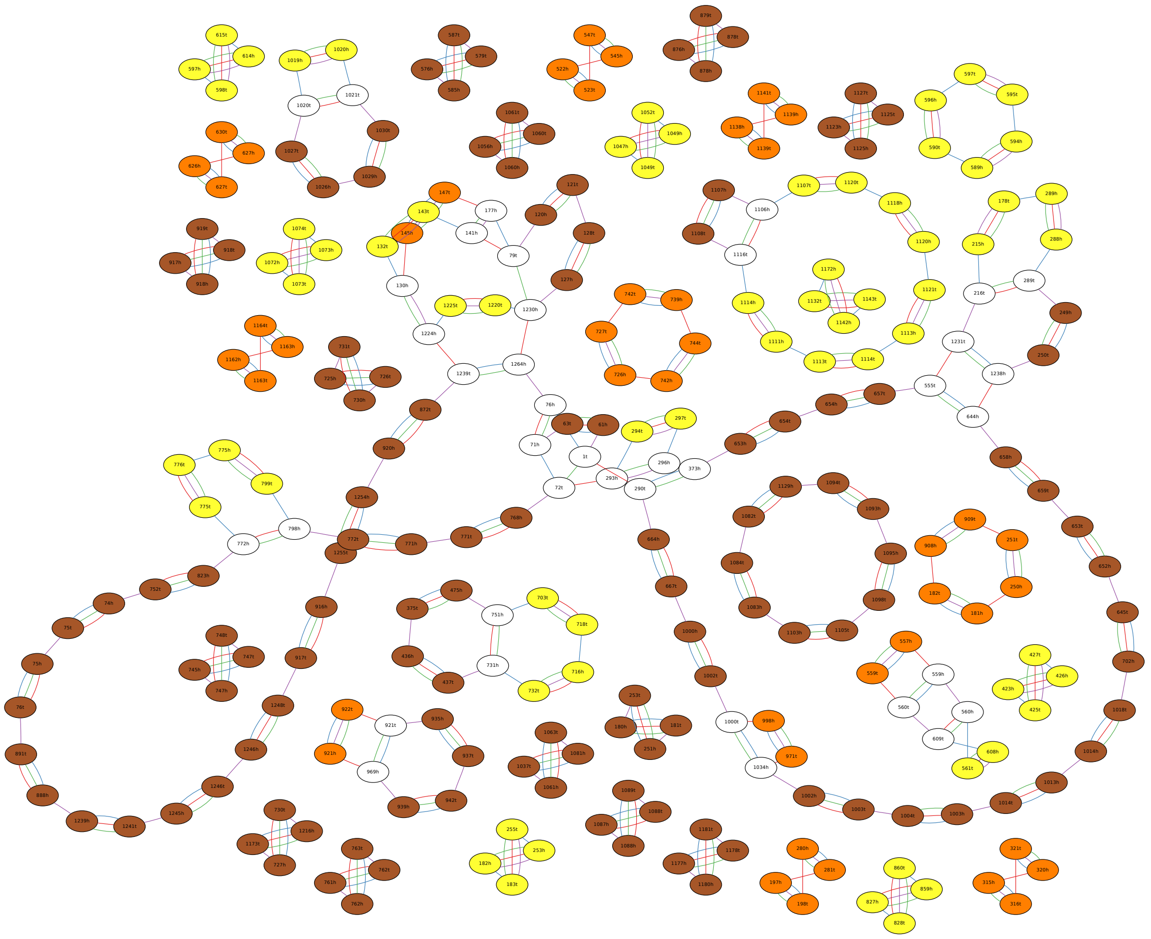

Figure S5: The multiple breakpoint graph [50] formed by genomes Q (red edges), H (blue edges), QH (green edges), and MRD (violet edges) on 433 synteny blocks larger than $500K$. Only 278 vertices that have two or more neighbors (representing breakpoints between the genomes) are shown. The breakpoints specific to only one breakpoint graph $G(Q, QH)$, $G(H, QH)$, or $G(MRD, QH)$ are colored in orange, yellow, or brown respectively.

	$Q+$	$H+$	$QH+$
$Q+$	44	6	14
$H+$		66	14
$QH+$			130

Table S4: The number of breakpoints specific to particular branches (entries on the diagonal) $H+$, $Q+$, and $QH+$ as well as to pairs of these branches (entries above the diagonal). The number of 3-way breakpoints (not included in the counts) is 4.

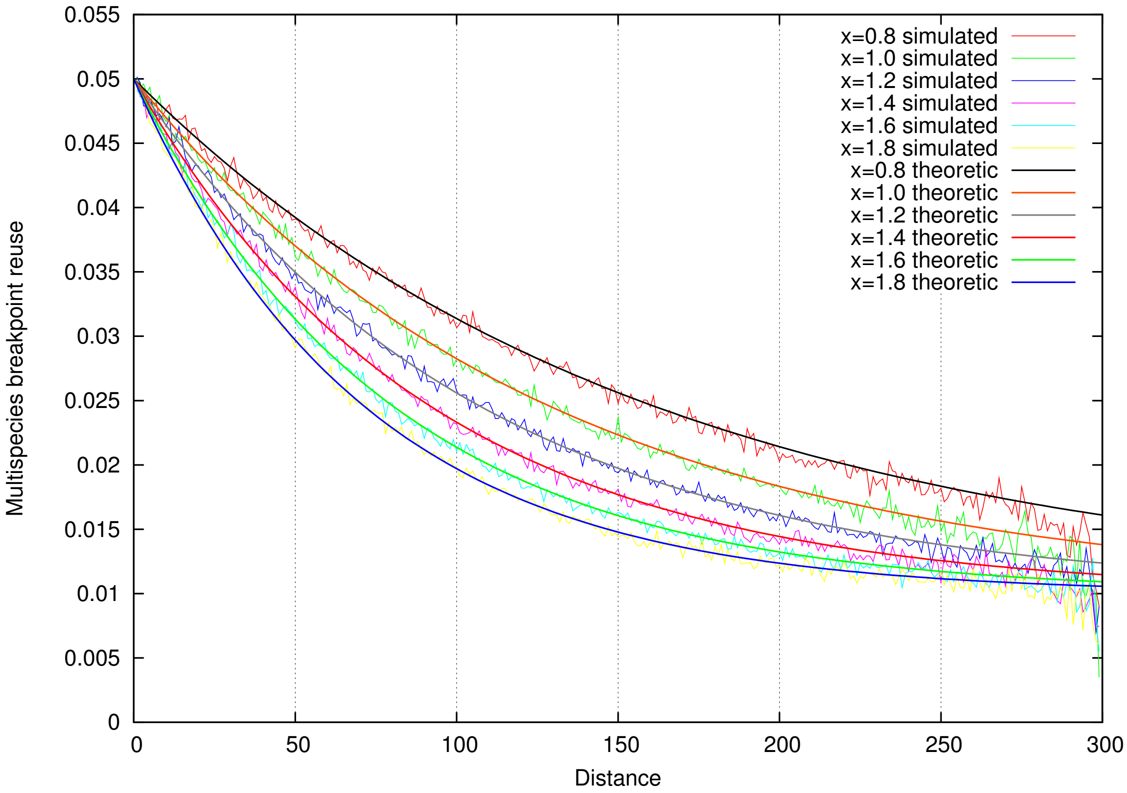

Figure S6: The multispecies breakpoint reuse $R(\ell)$ observed in simulation of 300 rearrangements under the TFBM model with $m = 800$, $n = 160$, and x ranging from 0.8 to 1.8 and the corresponding theoretical curves.
